# Surgery or General Medicine – a study of the reasons underlying the choice of medical specialty

**DOI:** 10.1590/S1516-31802004000300002

**Published:** 2004-05-06

**Authors:** Patrícia Lacerda Bellodi

**Keywords:** Career choice, Medical specialties, Medical education, Residents, Brazil, Profissão, Especialidades médicas, Educação médica, Brasil

## Abstract

**CONTEXT:**

The reality of medical services in Brazil points towards expansion and diversification of medical knowledge. However, there are few Brazilian studies on choosing a medical specialty.

**OBJECTIVE:**

To investigate and characterize the process of choosing the medical specialty among Brazilian resident doctors, with a comparison of the choice between general medicine and surgery.

**TYPE OF STUDY:**

Stratified survey.

**SETTING:**

Hospital das Clínicas, Faculdade de Medicina, Universidade de São Paulo (HC-FMUSP).

**METHODS:**

A randomized sample of resident doctors in general medicine (30) and surgery (30) was interviewed. Data on sociodemographic characteristics and the moment, stability and reasons for the choice of specialty were obtained.

**RESULTS:**

The moment of choice between the two specialties differed. Surgeons (30%) choose the specialty earlier, while general doctors decided progressively, mainly during the internship (43%). Most residents in both fields (73% general medicine, 70% surgery) said they had considered another specialty before the current choice. The main reasons for general doctors’ choice were contact with patients (50%), intellectual activities (30%) and knowledge of the field (27%). For surgeons the main reasons were practical intervention (43%), manual activities (43%) and the results obtained (40%). Personality was important in the choice for 20% of general doctors and for 27% of surgeons.

**DISCUSSION:**

The reasons found for the choice between general medicine and surgery were consistent with the literature. The concepts of wanting to be a general doctor or a surgeon are similar throughout the world. Personality characteristics were an important influencing factor for all residents, without statistical difference between the specialties, as was lifestyle. Remuneration did not appear as a determinant.

**CONCLUSION:**

The results from this group of Brazilian resident doctors corroborated data on choosing a medical specialty from other countries with different social and educational characteristics. This congruence indicates that the choice involves very similar desires and needs in different settings and has little dependence on the students’ educational context.

## RESUMO

**CONTEXTO:**

A realidade da Medicina no Brasil aponta para uma expansão e uma diversificação do conhecimento médico e uma variedade altamente especializada de serviços médicos. Entretanto há poucos estudos a respeito da escolha da especialidade médica em nosso país.

**OBJETIVO:**

Investigar e caracterizar o processo da escolha da especialidade médica em um grupo de residentes brasileiros, comparando a opção pela clínica médica e pela cirurgia.

**TIPO DE ESTUDO:**

pesquisa através de amostra estratificada e randomizada.

**LOCAL:**

Hospital das Clínicas da Faculdade de Medicina da Universidade de São Paulo (HCFMUSP).

**MÉTODOS:**

Há uma diferença significativa no momento da escolha entre as duas especialidades. Cirurgiões escolhem mais cedo a especialidade, antes mesmo do início do curso (30%), enquanto a decisão dos clínicos é progressiva ao longo do tempo, principalmente nos dois últimos anos, no internato (43%). A maioria dos residentes das duas especialidades (73% na clínica e 70% na cirurgia) chegou a considerar outra especialidade, mas mostrou-se bastante satisfeita com a escolha realizada. As principais razões de escolha pela clínica disseram respeito especialmente ao contato com o paciente (50%), ao gostar mais de atividades intelectuais (30%) e aos conhecimentos da área (27%). Para os cirurgiões, as principais razões de escolha disseram respeito ao tipo de intervenção prática e objetiva (43%), ao gostar de atividades manuais (43%) e aos resultados obtidos na área – rápidos e concretos (40%). A personalidade foi importante na escolha da especialidade para 20% dos clínicos e 27% dos cirurgiões.

**DISCUSSÃO:**

As razões da escolha pela clínica médica ou cirurgia neste estudo foram bem consistentes com os dados da literatura. Parece que os conceitos de “querer ser clínico” ou “querer ser cirurgião” são semelhantes no mundo. Características de personalidade foram fatores influenciadores importantes mencionados pelos residentes em geral, sem diferença estatística entre as especialidades. Entretanto, na análise qualitativa, a personalidade diferiu nas duas especialidades. Estilo de vida configurou-se da mesma forma. Remuneração não apareceu como determinante.

**CONCLUSÃO:**

Em geral, os resultados obtidos no Brasil reafirmam estudos realizados em outros países com características sociais e educacionais bastante diversas. Esta congruência indica que a escolha da especialidade médica é um processo vocacional que envolve desejos e necessidades muito similares e pouco dependente do contexto educacional do aluno.

## INTRODUCTION

At the moment of choosing a specialty, the medical student looks within himself and at the work possibilities. He searches for the option that will allow the best integration of these two worlds: the internal and the external. He thinks about who he is, what his interests are, what is important to him, what he could do well, and what the rewards that might be expected are. General medicine, surgery, pediatrics, psychiatry and others: there are many possibilities and these are becoming ever more numerous, due to the creation of subspecialties within the traditional specialties. Probably for this reason, one of the most frequent themes within medical education research relates to the choice of medical specialty, especially today, in view of the concern about the training and distribution of generalists.^1-8^

To understand this subject, several factors must be considered: sociodemographic data, the moment of choice, stability of the choice, academic aspects, factors of influence, personality characteristics and also opinions and perceptions regarding the various specialties.^1-8^ Especially in relation to the choice between the general medicine and surgical fields, the results in the literature have been quite consistent.^9-18^

In general, students cite the following reasons behind their decision to opt for general medicine: opportunity for a better degree and quality of contact with patients; patient type (e.g. elderly, chronic); opportunity for broad and comprehensive caregiving; diagnostic challenge; intellectual content; satisfaction in deepening the study of the patient; ambulatory practice; contact with practitioners in the area; the view that this kind of medical practice is the only one possible, because “to be in medicine is to be a general doctor”; opportunity to be involved in psychological and social aspects of medicine; desire to contribute to the community; need to keep options open; and finally, because they confer less value to aspects such as remuneration and lifestyle.^9-18^

The students that choose surgery justify their choice in terms of the opportunity for practical procedures and operations offered by the field of surgery; their enjoyment of emergency care; the effective results; the limiting of patients’ problems; the practical application of scientific knowledge; the research opportunities; the predominance of in-hospital practice; the prestige of this field within the medical profession; the opportunity for leadership; their desire to exercise authority; their greater interest in diagnosis and treatment than in interpersonal aspects of patient care; the greater remuneration; the lower degree of uncertainty in diagnosis; and the greater respect enjoyed by residents in this field.^9-18^

An important and extensive study^19^ of specialty profiles has shown that Brazil today recognizes 64 specialties. The major fields in which specialists were concentrated until 1997 were, in decreasing order: pediatrics, general surgery, general medicine, gynecology-obstetrics, anesthesiology, orthopedics, cardiology, psychiatry, ophthalmology and radiology. In the 1980s the most sought-after specialties were pediatrics, general medicine, gynecology-obstetrics, general surgery, cardiology, psychiatry, anesthesiology and orthopedics. Doctors receiving the greatest remuneration are concentrated in specialties such as occupational medicine, urology and plastic surgery.^19^

Men form the majority in some specialties, especially in the fields of surgery, orthopedics, urology and radiology. The presence of women is more pronounced in other areas such as pediatrics, general medicine, pathology, gynecology-obstetrics and psychiatry. Most of the specialists are young and in the 30 to 39-year age group.^19^

The reality of the medical services market in Brazil is pointing towards expansion and branching out of medical knowledge and a variety of highly specialized medical services already exist. The subspecialties originate from pediatrics, radiology, orthopedics and trauma-tology, plastic surgery, cardiology etc. They are increasingly common and constitute “microcosms of the process of medical care division.”^19^ However, despite this diversity, there are few studies concerning the choice of medical specialty in Brazil.

In a study involving final-year students at eight medical schools, the author^20^ verified that almost half of the students, when entering medical school, had already thought about a specialty. For one-quarter of these, their first choice prevailed. The students that preferred psychiatry and surgery presented the highest percentage of stability of choice. Differences appeared between men and women with regard to the reasons given for their choices. Men gave greater value to monetary income, immediate therapeutic results and having private work. Women, on the other hand, attributed more importance to an academic career and a work schedule that would be more regular.

Nearly half admitted to having experienced difficulties in their choice of specialty.

Another study^21^ comparing three groups of freshman students verified that there was considerable similarity among the students in the values that they attributed to the various specialties: the prestige associated with surgery and pediatrics; the profitability of surgery and psychiatry; the doctor-patient relationship in psychiatry and general practice; the intellectual capacity in general practice and pediatrics; and the possibility of controlling working hours in public health service and psychiatry.

A study^22^ investigating the relationship between gender and specialty choice found that, in Brazil, in comparison with other places in the world, there is still a significant difference between men and women in the choice of certain specialties. Women tend toward pediatrics and men toward surgery and orthopedics. There has been a decrease in the choice of general medicine for both sexes and an increase in the choice of anesthesiology among men and radiology among women.

Although the results found in Brazil are not generally discordant with those from other countries, there are, as already mentioned, very few national studies. Given the size of the country and the specific needs of the Brazilian healthcare system, the trends in specialty choice should be appraised continually.

This study aimed to investigate and characterize the process of choosing a medical specialty among a group of Brazilian resident doctors, especially with regard to comparing the choice between the fields of general medicine and surgery.

## METHODS

### Subjects

The participants were 60 residents in the first year (R1) and second year (R2) of the residence programs in surgery and general medicine of Hospital das Clínicas, Faculdade de Medicina, Universidade de São Paulo (HCFMUSP). Their distribution was as follows:

- General Medicine Group (Gen Med): 15 R1 (7 men and 8 women); 15 R2 (8 men and 7 women).
- Surgery Group (Surg): 15 R1 (10 men and 5 women); 15 R2 (11 men and 4 women).

In both the general medicine and surgery fields, the first two years of residence are in a general area, such that those who prefer may then choose a subspecialty.

The two groups were randomly constituted, with an attempt to compose a balanced sample with regard to sex. The final composition of each group was due to the gender characteristics of the total resident population in the two specialties. Surgery is still a predominantly masculine career field and, during the study period, there were only 10 women in the residence program, one of whom did not consent to participate in the research. In addition, two other residents, both in general medicine, did not agree to participate in the study, for personal reasons.

The residents as a whole were young, had entered university quite early (before the age of 20 years, on average), were single (only a few married women), Catholic in their majority, with parents in the same profession, and most of them came from the southeastern region of Brazil and from state capital cities. Surgery residents were alumni of public medical schools, especially FMUSP itself, while in the general medicine program there was a larger proportion of students from private schools.

## INSTRUMENT

The randomly selected residents that agreed to participate in the research were interviewed in sessions of approximately one hour.

The author developed a questionnaire, with open and closed questions, structured to include aspects considered important in the decision-making process for the choice of medical specialty. Data was obtained regarding sociodemographic characteristics, the moment of choice, stability of the choice and the reasons for the choice of specialty.

The answers obtained were classified and analyzed by the researcher herself, and by two other independent judges.

Data analysis and comparison between the variables studied were performed by means of non-parametric statistical analysis through the Mann Whitney U and chi-squared tests, adopting the significance level of p = 0.05.

## RESULTS

### The moment of choice

**Figure 1:**
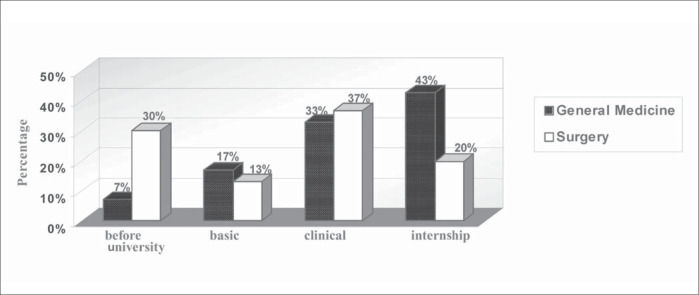
Moment of choice for the future field of work in medicine by medical residents in Hospital das Clínicas (São Paulo, Brazil).

There was a significant difference between the two specialties with regard to the moment of choice for the future field of work within medicine (Figure 1). It was observed that there is less movement among those that choose surgery: 30% of the surgery residents declared that they had chosen the specialty even before starting university, in comparison with only 7% of the general medicine residents. For those that choose general medicine the decision tends to consolidate as the course progresses: 43% of the general medicine residents, in comparison with only 20% of the surgeons, made the choice of specialty during the time of internship (p = 0.055).

### Stability of the choice

Although there was a difference in the moment of choice between the two specialties, most of the residents in both fields (73% in general medicine and 70% in surgery) declared that they had considered another specialty before the current choice (Table 1).

**Table 1:** Stability of the choice for the future field of work in medicine: other prior choices by medical residents in Hospital das Clínicas (São Paulo, Brazil)

	General medicine			Surgery		
Other Prior Choices	Male (n = 15) %	Fem. (n = 15) %	Total (n = 30) %	Male (n = 21) %	Fem. (n = 9) %	Total (n = 30) %
**NO**	20	33	**27**	29	33	**30**
**YES**	80	67	**73**	71	67	**70**
General surgery	47	27	**37**			
Neurology/neurosurgery	13	13	**13**			
Infectology		7	**3**			
Preventive medicine		7	**3**			
Radiology	7		**3**	5	11	**7**
Otorhinolaryngology	7		**3**	5		**3**
Dermatology		7	**3**		11	**3**
Pediatrics	13	20	**17**	10	11	**10**
Psychiatry	7		**3**	5	11	**7**
General medicine				19	11	**17**
Oncology				5		**3**
Orthopedics				19		**13**
Gynecology/obstetrics				10	11	**10**
Ophthalmology				5		**3**
Anesthesiology				5		**3**
Intensive therapy				10	11	**10**

***Note:** Categories are not mutually exclusive.*

It was observed that general doctors considered several other fields, while the surgeons considered fields where surgical techniques were also present (gynecology-obstetrics, ophthalmology, orthopedics etc).

### Reasons for the choice

The reasons given by the residents for their choice are shown in Table 2. The reasons indicated by general medicine residents were mainly the contact with patients (50%), intellectual activities (30%) and knowledge of the field (27%). When their reasons were compared with those presented by the surgery residents, statistically significant differences were found in the following categories (illustrated with some examples):

**Table 2:** Reasons for the choice for the future field of work in Medicine by medical residents in Hospital das Clínicas (São Paulo, Brazil)

	General edicine			Surgery		
Why This Speciality?	Male (n = 15) %	Fem. (n = 15) %	Total (n = 30) %	Male (n = 21) %	Fem. (n = 9) %	Total (n = 30) %
Contact with patient *	40	60	**50**			
Overall view of patient *	20	27	**23**			
Knowledge of the field	27	27	**27**	10	11	**10**
Results obtained *				33	56	**40**
Preference for intellectual activities *	40	20	**30**			
Preference for manual activities *				38	56	**43**
I have manual dexterity					11	**3**
I do not have manual dexterity	7	7	**7**			
Type of intervention (continual and preventive)*	13	13	**13**			
Type of intervention (practical and objective) *				52	22	**43**
Because of lifestyle	20	27	**23**	10		**7**
Because of the diversity of fields	7		**3**	10		**7**
By exclusion, lack of option *		27	**13**			
Type of patient (acute problems) *				14	11	**13**
Type of patient (chronic problems)		13	**7**			
Surgeon is a complete doctor *				14	22	**17**
Clinical doctor is “the true doctor” *		27	**13**			
Type of personality	7	33	**20**	29	22	**27**
Family influence	7	7	**7**	5		**3**
Opposition to family influence	7		**3**			
Influence of others					11	**3**
Identification with a role-model at the medical school		7	**3**		22	**7**
Non-parametric Mann-Whitney U test (p < 0.05)						

**Note**: *Categories are not mutually exclusive.*

* *Statistically significant difference between the specialties. Intra-group comparison between sexes: Clinical (male = female) – for all reasons; surgery (male = female) – for all reasons.*

- Because of the contact with patients (p = 0.000)
  *“I am fascinated by the doctor-patient relationship; the choice could not be a specialty without a patient…”* (Gen Med male)
- Because of the overall view of the patient (p = 0.005)
  *“In order to have the possibility of seeing the patient in an integrated manner…”* (Gen Med male) • Because of preference for intellectual activities (p = 0.001)
  *“Because I like general medicine. I really enjoy clinical reasoning; it gives me pleasure…”* (Gen Med male).
- Because of the type of intervention (continuous preventive) (p = 0.040)
  *“The general doctor follows the patient up continuously. Chronic disease creates a tie…”* (Gen Med female).
- By elimination, because of a lack of choice (p = 0.040)
  *“I knew what I didn't want. I never liked surgery; I don't like procedures…”* (Gen Med female).
- Because the general doctor is the “true doctor”; general medicine is “what medicine is about” (p = 0.040)
  *“I thought being a doctor would be like this: working with reasoning and hypotheses…”* (Gen Med male).

For surgery residents, the most important reasons related to the practical and objective aspects of surgical intervention (43%), the manual activities (43%) and the results obtained (40%).

The following aspects were significant for residents of the surgery area (followed by examples):

- Because of preference for manual activities (p = 0.000)
  *“I like to manipulate. The general doctor thinks he cures, but he doesn't put his hands on the patient to help…”* (Surg male).
- Because of the type of surgical intervention (practical/objective) (p = 0.000)
  *“The possibility of being practical, objective. If you can't operate, then acquire patience…”* (Surg male).
- Because of the results obtained (fast, visible and effective) (p = 0.000)
  *“Because you can see things happening through what you are doing. You can see that a tumor is being removed…”* (Surg male).
- Because the surgeon is the complete doctor; surgery goes beyond general medicine (p = 0.021)
  *“He would be, modesty aside… a more complete doctor: he is a general doctor and also a surgeon”* (Surg male).
- Because of the type of patient (acute problems) (p = 0.040)
  *“I don't like chronic patients. [On the other hand, through surgery] a young patient goes into the hospital and leaves well, even if the problem was acute…”* (Surg male).

It is important to highlight that there were no statistically significant differences between men and women within each specialty, in relation to the reasons for the choice that were presented.

## DISCUSSION

The general medicine residents’ main reasons involved essentially two basic aspects of this clinical field: the subjective relationship with the patient and cognitive knowledge. Some residents summarized these two fundamental aspects of general medicine in their responses to justify their choice, for instance: *“It stimulates more research and has a closer relationship with the patient. I enjoy creating a stronger bond with the patient. The length of time you see the patient in the other fields is very short… the field of knowledge is more wide-ranging: you have to know at least a little of everything. I don't want to be a super-specialist in just one thing…”* (Gen Med male).

Likewise, the surgeons’ main reasons were related to the fundamental aspects of the field of surgery: the technical abilities inherent to surgical activity and the less direct and less personal doctor-patient relationship, which tends to be standardized and usually programmed. Some answers among the residents of surgery condensed these aspects, for instance: *“I like to cut and sew. Because of the type of patient, I would never want to get into geriatrics or neurology. I prefer young people; cases that are resolved more smoothly. He/she comes and is operated on: surgery gives the impression of a more tangible thing, work with a faster result.”* (Surg male).

For the surgeons, their personality traits attracted them positively to that specialty. For some of the general doctors, their personal characteristics did not so much favor entering the field of general medicine, but rather they steered these doctors away from surgery. The general doctors stated in relation to personality: *“I did not have a personality profile for surgery, nor did I have manual dexterity. I found surgery tedious…”* (Gen Med male).

On the other hand, surgery residents stated, in relation to their personality characteristics, that: *“It has to do with personality. I am a practical guy and I don't like beating about the bush: the problem is this and it has to be solved in this way…”* (Surg male).

The surgeon's lifestyle was responsible for some general doctors’ decision against the field of surgery, as expressed in their responses. There was a desire to be a surgeon, but not the desire to work as one: *“Because of the lifestyle of surgery, without scheduling for anything, without a set time to eat lunch: the surgeon doesn't know when it will finish. It's a lot of hours on your feet…”* (Gen Med male).

When abilities and personality were considered as internal determinants, they supplanted the external ones (influence of others in the socialization process, social representation of the field and lifestyle), for residents in the fields of both general medicine and surgery.

However, internal determinants as a whole were more important in the surgeons’ choice than in the general doctors’ choice. While 57% of the residents in general medicine cited abilities and personality as determinant factors, the proportion for residents in surgery was approximately 73%. For the general doctors, external determinants prevailed, especially lifestyle, which was mentioned more by the general doctors (23%) than by the surgeons (7%).

It is interesting to note at this point that remuneration, although mentioned in the literature as quite important in the decision to specialize in surgery, did not appear as a determinant in Brazil, either for the choice of general medicine or for the choice of surgery.

To conclude the discussion regarding the factors influencing the choice of each specialty, it is worth commenting on the opinions and perceptions showed by the residents. As has been described in the literature, medical students choose the field of general medicine by “considering that this approach to medicine is the only one that can be practiced and, therefore, to be a doctor is to be a general doctor”. Some residents in the present study also justified their choice with the response: “because the general doctor is the ‘true doctor’; general medicine is ‘what medicine is about’”. This was sometimes associated with their doctor during childhood: *“In the child's ideal, the doctor is the general doctor”* (Gen Med female).

Although the justification among surgery residents in the present study that “the surgeon is a complete doctor; surgery goes beyond general medicine” did not appear among the most frequent reasons indicated in the literature, this seems to lead on to another aspect that is notably present in several studies: omnipotence, arrogance and narcissism as surgeons’ personality characteristics. *“It's like winning a game or a contest … There is some power in performing surgery: this gives pleasure. You go there and it gets done. The difficult part is the best part. The surgeon solves the situation. We can do clinical treatment: no need to call the other guy, the general doctor. A surgeon is more independent, more complete. Like a general practitioner, he is more complete…”* (Surg male).

## CONCLUSIONS

In summary, the results regarding the process of choosing a medical specialty among a group of Brazilian residents corroborated data found in other studies on this topic.

This congruence indicates that becoming a doctor – general doctor or surgeon – is a process with very similar questions, desires and needs in different places in the world and has little dependence on the student's educational context.
